# Supplementary material for: Leveraging cancer mutation data to inform the pathogenicity classification of germline missense variants
Source: PLoS Genet. 2025 Jan 6;21(1):e1011540. doi: 10.1371/journal.pgen.1011540 (PMC11737861; doi:10.1371/journal.pgen.1011540)
Supplement: S1 Text — (DOCX) [file pgen.1011540.s001.docx]

**SUPPLEMENTAL FILE FOR:** Leveraging cancer mutation data to inform the pathogenicity classification of germline missense variants

**Supplemental Methods**

***Identifying functional cancer evidence for mutations from Cancer Hotspots***

We searched for mutations in cancer genes sourced from Cancer Hotspots (n = 216) in the Jackson Laboratory Clinical Knowledgebase (CKB) database [1]. In CKB, the protein-level effect of each mutation is categorized as "loss of function," "gain of function," "no effect," or "unknown", based on manual curation of the published literature. We did not assess whether the functional evidence from CKB aligns with ClinGen recommendations for a "well-established" functional assay to qualify for the PS3 criterion [2].

***Applying lines of evidence for germline variant classification***

We calculated the positive likelihood ratio (LR+) that assesses the degree to which a missense variant being present in the Cancer Hotspot database increases the odds that the germline variant is classified as likely pathogenic/pathogenic (LP/P) in ClinVar [3]. The LR+ was determined using the following equation:

$${LR}^{+}=\frac{Sensitivity}{1- Specificity)}=\frac{\frac{TP}{(TP+FN)}}{1- \frac{TN}{(TN+FP)}}$$

where “true positive” (TP) represents the number of ClinVar LP/P variants that overlap with cancer mutations in Cancer Hotspots, “false negative” (FN) represents ClinVar likely pathogenic/pathogenic (LP/P) variants that are absent in Cancer Hotspots, “true negative” (TN) represents the number of ClinVar non-LP/P variants in the same gene set that are absent in Cancer Hotspots, and “false positive” (FP) represents the number of ClinVar non-LP/P variants in the same gene set that are overlap with cancer mutations in Cancer Hotspots. LR+ values were compared with the corresponding ACMG/AMP evidence strengths for pathogenic evidence reported in Tavtigian et al. (2018). The paucity of likely benign/benign (LB/B) variants in ClinVar that overlapped with Cancer Hotspots risked inflating the LR+ calculation (Supplemental Table 4), and so we also considered all variants of uncertain significance (VUS) and with conflicting interpretations of pathogenicity (CIP) in ClinVar as LB/B as an extreme conservative step to lower bound the LR+.

For each VUS in ClinVar that overlapped with Cancer Hotspots, we determined the ACMG/AMP variant classification evidence codes that could be applied in the absence of clinical/family data (e.g., PM2_Supporting, PP2, PP3, PP3_Moderate, and PP3_Strong). The ACMG/AMP combining rules were then used to clarify whether additional evidence from the cancer LR+ could hypothetically upgrade VUS classifications to LP/P.

***Plotting distribution of REVEL and AlphaMissense scores***

The REVEL and AlphaMissense scores were obtained for the ClinVar dataset (pre-filtering) [4,5]. We plotted the distribution of both scores for germline variants in ClinVar that overlapped with cancer mutations in Cancer Hotspots (CH+, ClinVar+), germline variants that do not overlap with cancer mutations (CH-, ClinVar+), and all remaining cancer mutations that do not overlap with germline variants (CH+, ClinVar-). The median score was calculated for each group.

***Exploratory analysis with independent variables prior to model training***

We performed an analysis of all independent variables to evaluate their overall impact on the ClinVar dataset (pre-filtering). Specifically, we focused on a subset of eight genes (*TP53*, *PIK3CA*, *PTEN*, *SMAD4*, *VHL*, *PTPN11*, *RIT1*, and *FGFR3*), plotting their total number of cancer mutations from Cancer Hotspots. The inclusion of the proportion of these mutations overlapping with germline variants in ClinVar allowed us to discern variations in LP/P variant enrichment across these genes, highlighting their relative pathogenicity within the model.

We also evaluated the ability of tumor sample counts from CH cancer mutations overlapping with ClinVar germline variants to differentiate between LP/P and LB/B/VUS classifications. Plotting these values by ClinVar classification groups (LP/P and LB/B/VUS), we generated receiver operating characteristic (ROC) curves and compared area under the curve (AUC) values to determine the tumor sample count cut-off that best discriminates between the two groups of classifications. We found this cut-off to be >25 tumour sample counts, which also influences predicted pathogenicity scores for the LRM, and represents a cut-off for a node in RFM decision trees by splitting the training data with the lowest Gini impurity.

Additionally, we generated four plots to visualize phyloP and phastCons scores (both mammalian and vertebrate scores). Each plot displayed the distribution of LP/P and VUS variants for the CH+, ClinVar+ and CH-, ClinVar+ variant groups. We compared these variant groups (CH+, ClinVar+ versus CH-, ClinVar+) by median scores and computing the probability of superiority (PS), to evaluate the relative superiority of values between the two groups.

***Investigating overlap of non-coding cancer mutations from COSMIC with germline variants***

Non-coding cancer mutations (n = 434,213) in GRCh37 were downloaded from the COSMIC database [6] (release date: May 2023) and annotated using a custom pipeline developed by The Centre for Applied Genomics (TCAG) in Toronto, Canada. ClinVar annotations (date accessed: Sep 2023) were used to identify mutations that were observed as germline variants along with their classifications. However, we observed a modest degree of overlap and we were underpowered to employ similar methods as for the missense mutations from Cancer Hotspots. In many cases, ClinVar classifications for variants corresponded to the coding region of an alternate transcript, rather than the non-coding region where cancer mutation was observed.

***Evaluating the impact of conservation scores on supervised learning models***

Logistic regression and random forest models were generated using only conservation scores as independent variables to investigate whether their inclusion substantially impacts classification outcomes (LP/P versus LB/B). Additionally, logistic regression and random forest models were generated without conservation scores as independent variables and their performance was evaluated on the test dataset using area under the precision-recall curve (AUPRC) and area under the receiver-operating characteristic curve (AUROC). This comparison was completed to demonstrate that the exclusion of conservation scores reduced model performance, while their inclusion enhanced model fit and convergence by providing continuous independent variables that would otherwise be missing.

**Supplemental References**

 1. Patterson SE, Liu R, Statz CM, Durkin D, Lakshminarayana A, Mockus SM. The clinical trial landscape in oncology and connectivity of somatic mutational profiles to targeted therapies. Hum Genomics. 2016;10: 4. doi:10.1186/s40246-016-0061-7

2. Brnich SE, Abou Tayoun AN, Couch FJ, Cutting GR, Greenblatt MS, Heinen CD, et al. Recommendations for application of the functional evidence PS3/BS3 criterion using the ACMG/AMP sequence variant interpretation framework. Genome Medicine. 2019;12: 3. doi:10.1186/s13073-019-0690-2

3. Tavtigian SV, Greenblatt MS, Harrison SM, Nussbaum RL, Prabhu SA, Boucher KM, et al. Modeling the ACMG/AMP variant classification guidelines as a Bayesian classification framework. Genet Med. 2018;20: 1054–1060. doi:10.1038/gim.2017.210

4. Ioannidis NM, Rothstein JH, Pejaver V, Middha S, McDonnell SK, Baheti S, et al. REVEL: An Ensemble Method for Predicting the Pathogenicity of Rare Missense Variants. Am J Hum Genet. 2016;99: 877–885. doi:10.1016/j.ajhg.2016.08.016

5. Cheng J, Novati G, Pan J, Bycroft C, Žemgulytė A, Applebaum T, et al. Accurate proteome-wide missense variant effect prediction with AlphaMissense. Science. 2023;381: eadg7492. doi:10.1126/science.adg7492

6. Tate JG, Bamford S, Jubb HC, Sondka Z, Beare DM, Bindal N, et al. COSMIC: the Catalogue Of Somatic Mutations In Cancer. Nucleic Acids Research. 2019;47: D941–D947. doi:10.1093/nar/gky1015
