# Supplementary material for: Leveraging cancer mutation data to inform the pathogenicity classification of germline missense variants
Source: PLoS Genet. 2025 Jan 6;21(1):e1011540. doi: 10.1371/journal.pgen.1011540 (PMC11737861; doi:10.1371/journal.pgen.1011540)
Supplement: S2 Text — Supplemental Tables A-E. (DOCX) [file pgen.1011540.s002.docx]

**SUPPLEMENTAL FILE FOR:** Leveraging cancer mutation data to inform the pathogenicity classification of germline missense variants

**TABLE OF CONTENTS**

Table A…………………………………………………………………………………...….…2

Table B…………………………………………………………………………………...….…3

Table C…………………………………………………………………………………...….…4

Table D…………………………………………………………………………………...….…5

Table E…………………………………………………………………………………...….…6

Table A. Genes with missense mutations from Cancer Hotspots that overlap with germline variants in ClinVar, along with their OMIM disease phenotype(s).

| **Gene** | **Cancer Gene Type** | **Phenotype Classification** | **OMIM Disease Phenotype** |
| --- | --- | --- | --- |
| *ACVR1* | Proto-oncogene | Multisystem phenotype | Fibrodysplasia ossificans progressiva, 135100 (3), i:AD |
| *AKT1* | Proto-oncogene | Multisystem phenotype | Breast cancer, somatic, 114480 (3); Cowden syndrome 6, 615109 (3); Proteus syndrome, somatic, 176920 (3); Ovarian cancer, somatic, 167000 (3); Colorectal cancer, somatic, 114500 (3) |
| *ALK* | Oncogene, fusion | Hereditary cancer predisposition phenotype | {Neuroblastoma, susceptibility to, 3}, 613014 (3) |
| *APC* | Tumour suppressor gene | Hereditary cancer predisposition phenotype | Adenoma, periampullary, somatic, 175100 (3); Desmoid disease, hereditary, 135290 (3), i:AD; Adenomatous polyposis coli, 175100 (3), i:AD; Gardner syndrome, 175100 (3), i:AD; Hepatoblastoma, somatic, 114550 (3); Colorectal cancer, somatic, 114500 (3); Gastric adenocarcinoma and proximal polyposis of the stomach, 619182 (3), i:AD; Brain tumor-polyposis syndrome 2, 175100 (3), i:AD; Gastric cancer, somatic, 613659 (3) |
| *ATM* | Tumour suppressor gene | Multisystem phenotype/Hereditary cancer predisposition phenotype | Lymphoma, mantle cell, somatic (3); Lymphoma, B-cell non-Hodgkin, somatic (3); Ataxia-telangiectasia, 208900 (3), i:AR; {Breast cancer, susceptibility to}, 114480 (3), i:AD, i:SMu; T-cell prolymphocytic leukemia, somatic (3) |
| *BRAF* | Oncogene, fusion | Multisystem phenotype | Nonsmall cell lung cancer, somatic (3); Noonan syndrome 7, 613706 (3), i:AD; Cardiofaciocutaneous syndrome, 115150 (3), i:AD; Colorectal cancer, somatic, 114500 (3); Adenocarcinoma of lung, somatic, 211980 (3); Melanoma, malignant, somatic,, 155600 (3); LEOPARD syndrome 3, 613707 (3), i:AD |
| *BRCA2* | Tumour suppressor gene | Hereditary cancer predisposition phenotype | {Pancreatic cancer 2}, 613347 (3); {Breast cancer, male, susceptibility to}, 114480 (3), i:AD, i:SMu; {Glioblastoma 3}, 613029 (3), i:AR; Wilms tumor, 194070 (3), i:AD, i:SMu; Fanconi anemia, complementation group D1, 605724 (3), i:AR; {Medulloblastoma}, 155255 (3), i:AD, i:AR, i:SMu; {Prostate cancer}, 176807 (3), i:AD, i:SMu; {Breast-ovarian cancer, familial, 2}, 612555 (3), i:AD |
| *CARD11* | Proto-oncogene | Multisystem phenotype | Immunodeficiency 11B with atopic dermatitis, 617638 (3), i:AD; B-cell expansion with NFKB and T-cell anergy, 616452 (3), i:AD; Immunodeficiency 11A, 615206 (3), i:AR |
| *CASP8* | Tumour suppressor gene | Multisystem phenotype | {Lung cancer, protection against}, 211980 (3), i:AD, i:SMu; ?Autoimmune lymphoproliferative syndrome, type IIB, 607271 (3), i:AR; Hepatocellular carcinoma, somatic, 114550 (3); {Breast cancer, protection against}, 114480 (3), i:AD, i:SMu |
| *CDH1* | Tumour suppressor gene | Multisystem phenotype/Hereditary cancer predisposition phenotype | Endometrial carcinoma, somatic, 608089 (3); {Prostate cancer, susceptibility to}, 176807 (3), i:AD, i:SMu; Blepharocheilodontic syndrome 1, 119580 (3), i:AD; Gastric cancer, hereditary diffuse, with or without cleft lip and/or palate, 137215 (3), i:AD; {Breast cancer, lobular}, 114480 (3), i:AD, i:SMu; Ovarian cancer, somatic, 167000 (3) |
| *CDK4* | Proto-oncogene | Hereditary cancer predisposition phenotype | {Melanoma, cutaneous malignant, 3}, 609048 (3), i:AD |
| *CDKN1B* | Tumour suppressor gene | Hereditary cancer predisposition phenotype | Multiple endocrine neoplasia, type IV, 610755 (3), i:AD |
| *CDKN2A* | Tumour suppressor gene | Hereditary cancer predisposition phenotype | {Melanoma and neural system tumor syndrome}, 155755 (3), i:AD; {Melanoma, cutaneous malignant, 2}, 155601 (3), i:AD; {Melanoma-pancreatic cancer syndrome}, 606719 (3), i:AD |
| *CHEK2* | Tumour suppressor gene | Hereditary cancer predisposition phenotype | Li-Fraumeni syndrome, 609265 (3); Osteosarcoma, somatic, 259500 (3); {Prostate cancer, familial, susceptibility to}, 176807 (3), i:AD, i:SMu; {Breast cancer, susceptibility to}, 114480 (3), i:AD, i:SMu; {Breast and colorectal cancer, susceptibility to} (3) |
| *CREBBP* | oncogene, TSG, fusion | Multisystem phenotype | Rubinstein-Taybi syndrome 1, 180849 (3), i:AD; Menke-Hennekam syndrome 1, 618332 (3), i:AD |
| *CTCF* | Tumour suppressor gene | Multisystem phenotype | Mental retardation, i:AD 21, 615502 (3), i:AD |
| *CTLA4* | Oncogene(?) | Multisystem phenotype | {Systemic lupus erythematosus, susceptibility to}, 152700 (3), i:AD; {Diabetes mellitus, insulin-dependent, 12}, 601388 (3); {Celiac disease, susceptibility to, 3}, 609755 (3); Autoimmune lymphoproliferative syndrome, type V, 616100 (3), i:AD; {Hashimoto thyroiditis}, 140300 (3), i:AD |
| *CTNNB1* | Oncogene, fusion | Multisystem phenotype | Ovarian cancer, somatic, 167000 (3); Colorectal cancer, somatic, 114500 (3); Medulloblastoma, somatic, 155255 (3); Hepatocellular carcinoma, somatic, 114550 (3); Pilomatricoma, somatic, 132600 (3); Neurodevelopmental disorder with spastic diplegia and visual defects, 615075 (3), i:AD; Exudative vitreoretinopathy 7, 617572 (3), i:AD |
| *DICER1* | Tumour suppressor gene | Hereditary cancer predisposition phenotype | GLOW syndrome, somatic mosaic, 618272 (3); Rhabdomyosarcoma, embryonal, 2, 180295 (3); Goiter, multinodular 1, with or without Sertoli-Leydig cell tumors, 138800 (3), i:AD; Pleuropulmonary blastoma, 601200 (3), i:AD |
| *DNMT1* | N/A | Multisystem phenotype | Cerebellar ataxia, deafness, and narcolepsy, i:AD, 604121 (3), i:AD; Neuropathy, hereditary sensory, type IE, 614116 (3), i:AD |
| *DNMT3A* | Tumour suppressor gene | Multisystem phenotype | Heyn-Sproul-Jackson syndrome, 618724 (3), i:AD; Acute myeloid leukemia, somatic, 601626 (3); Tatton-Brown-Rahman syndrome, 615879 (3), i:AD |
| *EGFR* | Proto-oncogene | Multisystem phenotype | ?Inflammatory skin and bowel disease, neonatal, 2, 616069 (3), i:AR; Nonsmall cell lung cancer, response to tyrosine kinase inhibitor in, 211980 (3), i:AD, i:SMu; Adenocarcinoma of lung, response to tyrosine kinase inhibitor in, 211980 (3), i:AD, i:SMu; {Nonsmall cell lung cancer, susceptibility to}, 211980 (3), i:AD, i:SMu |
| *ERBB2* | Oncogene, fusion | No OMIM Mendelian disorder | Glioblastoma, somatic, 137800 (3); Adenocarcinoma of lung, somatic, 211980 (3); Gastric cancer, somatic, 613659 (3); Ovarian cancer, somatic (3) |
| *ETV6* | Tumour suppressor gene, fusion | Multisystem phenotype | Leukemia, acute myeloid, somatic, 601626 (3); Thrombocytopenia 5, 616216 (3), i:AD |
| *FBXW7* | Tumour suppressor gene | Multisystem phenotype | [common variants in Wilms tumor; neurodevelopmental phenotype with variable features, hypotonia and constipation] |
| *FGFR1* | Oncogene, fusion | Multisystem phenotype | Pfeiffer syndrome, 101600 (3), i:AD; Jackson-Weiss syndrome, 123150 (3), i:AD; Trigonocephaly 1, 190440 (3), i:AD; Hypogonadotropic hypogonadism 2 with or without anosmia, 147950 (3), i:AD; Hartsfield syndrome, 615465 (3), i:AD; Osteoglophonic dysplasia, 166250 (3), i:AD; Encephalocraniocutaneous lipomatosis, somatic mosaic, 613001 (3) |
| *FGFR2* | Oncogene, fusion | Multisystem phenotype | Apert syndrome, 101200 (3), i:AD; Craniosynostosis, nonspecific (3); Jackson-Weiss syndrome, 123150 (3), i:AD; Scaphocephaly and Axenfeld-Rieger anomaly (3); Saethre-Chotzen syndrome, 101400 (3), i:AD; Gastric cancer, somatic, 613659 (3); Scaphocephaly, maxillary retrusion, and mental retardation, 609579 (3); Bent bone dysplasia syndrome, 614592 (3), i:AD; LADD syndrome, 149730 (3), i:AD; Craniofacial-skeletal-dermatologic dysplasia, 101600 (3), i:AD; Pfeiffer syndrome, 101600 (3), i:AD; Crouzon syndrome, 123500 (3), i:AD; Beare-Stevenson cutis gyrata syndrome, 123790 (3), i:AD; Antley-Bixler syndrome without genital anomalies or disordered steroidogenesis, 207410 (3), i:AD |
| *FGFR3* | Oncogene, fusion | Multisystem phenotype | Muenke syndrome, 602849 (3), i:AD; Nevus, epidermal, somatic, 162900 (3); Thanatophoric dysplasia, type II, 187601 (3), i:AD; Bladder cancer, somatic, 109800 (3); CATSHL syndrome, 610474 (3), i:AD, i:AR; Crouzon syndrome with acanthosis nigricans, 612247 (3), i:AD; Hypochondroplasia, 146000 (3), i:AD; LADD syndrome, 149730 (3), i:AD; Achondroplasia, 100800 (3), i:AD; Thanatophoric dysplasia, type I, 187600 (3), i:AD; Colorectal cancer, somatic, 114500 (3); Spermatocytic seminoma, somatic, 273300 (3); Cervical cancer, somatic, 603956 (3); SADDAN, 616482 (3), i:AD |
| *FGFR4* | Proto-oncogene | Multisystem phenotype/Hereditary cancer predisposition phenotype | {Cancer progression/metastasis} (3) |
| *FOXP1* | Oncogene, fusion | Multisystem phenotype | Mental retardation with language impairment and with or without autistic features, 613670 (3), i:AD |
| *GATA2* | Proto-oncogene | Multisystem phenotype/Hereditary cancer predisposition phenotype | Emberger syndrome, 614038 (3), i:AD; {Myelodysplastic syndrome, susceptibility to}, 614286 (3); Immunodeficiency 21, 614172 (3), i:AD; {Leukemia, acute myeloid, susceptibility to}, 601626 (3), i:AD, i:SMu |
| *GNAQ* | Proto-oncogene | Multisystem phenotype | Sturge-Weber syndrome, somatic, mosaic, 185300 (3); Capillary malformations, congenital, 1, somatic, mosaic, 163000 (3) |
| *GNAS* | Proto-oncogene | Multisystem phenotype | ACTH-independent macronodular adrenal hyperplasia, 219080 (3), i:SMu; Pseudohypoparathyroidism Ic, 612462 (3), i:AD; Pseudohypoparathyroidism Ib, 603233 (3), i:AD; Pseudopseudohypoparathyroidism, 612463 (3), i:AD; McCune-Albright syndrome, somatic, mosaic, 174800 (3); Osseous heteroplasia, progressive, 166350 (3), i:AD; Pituitary adenoma 3, multiple types, somatic, 617686 (3); Pseudohypoparathyroidism Ia, 103580 (3), i:AD |
| *HNF1A* | Tumour suppressor gene | Multisystem phenotype | {Diabetes mellitus, insulin-dependent}, 222100 (3), i:AR; MODY, type III, 600496 (3), i:AD; Hepatic adenoma, somatic, 142330 (3); Renal cell carcinoma, 144700 (3); Diabetes mellitus, insulin-dependent, 20, 612520 (3); {Diabetes mellitus, noninsulin-dependent, 2}, 125853 (3), i:AD |
| *HRAS* | Proto-oncogene | Multisystem phenotype | Nevus sebaceous or woolly hair nevus, somatic, 162900 (3); Congenital myopathy with excess of muscle spindles, 218040 (3), i:AD; Bladder cancer, somatic, 109800 (3); Thyroid carcinoma, follicular, somatic, 188470 (3); Schimmelpenning-Feuerstein-Mims syndrome, somatic mosaic, 163200 (3); Spitz nevus or nevus spilus, somatic, 137550 (3); Costello syndrome, 218040 (3), i:AD |
| *IDH1* | Proto-oncogene | Multisystem phenotype | {Glioma, susceptibility to, somatic}, 137800 (3) |
| *IDH2* | Proto-oncogene | Multisystem phenotype | D-2-hydroxyglutaric aciduria 2, 613657 (3) |
| *IL7R* | Proto-oncogene | Multisystem phenotype | Severe combined immunodeficiency, T-cell negative, B-cell/natural killer cell-positive type, 608971 (3), i:AR |
| *KDR* | Proto-oncogene | Multisystem phenotype | {Hemangioma, capillary infantile, susceptibility to}, 602089 (3), i:AD; Hemangioma, capillary infantile, somatic, 602089 (3) |
| *KIT* | Proto-oncogene | Hereditary cancer predisposition phenotype | Gastrointestinal stromal tumor, familial, 606764 (3), i:AD, Isolated cases; Mastocytosis, cutaneous, 154800 (3), i:AD; Germ cell tumors, somatic, 273300 (3); Leukemia, acute myeloid, somatic, 601626 (3); Mastocytosis, systemic, somatic, 154800 (3); Piebaldism, 172800 (3), i:AD |
| *KMT2D* | Oncogene, tumour suppressor gene | Multisystem phenotype | Kabuki syndrome 1, 147920 (3), i:AD |
| *KRAS* | Proto-oncogene | Multisystem phenotype | Oculoectodermal syndrome, somatic, 600268 (3); Leukemia, acute myeloid, somatic, 601626 (3); Breast cancer, somatic, 114480 (3); RAS-associated autoimmune leukoproliferative disorder, 614470 (3), i:AD; Cardiofaciocutaneous syndrome 2, 615278 (3), i:AD; Arteriovenous malformation of the brain, somatic, 108010 (3); Bladder cancer, somatic, 109800 (3); Pancreatic carcinoma, somatic, 260350 (3); Lung cancer, somatic, 211980 (3); Gastric cancer, somatic, 137215 (3); Schimmelpenning-Feuerstein-Mims syndrome, somatic mosaic, 163200 (3); Noonan syndrome 3, 609942 (3), i:AD |
| *MAP2K1* | Proto-oncogene | Multisystem phenotype | Cardiofaciocutaneous syndrome 3, 615279 (3), i:AD; Melorheostosis, isolated, somatic mosaic, 155950 (3) |
| *MAP2K2* | Proto-oncogene | Multisystem phenotype | Cardiofaciocutaneous syndrome 4, 615280 (3), i:AD |
| *MAX* | Tumour suppressor gene | Hereditary cancer predisposition phenotype | {Pheochromocytoma, susceptibility to}, 171300 (3), i:AD |
| *MET* | Proto-oncogene | Hereditary cancer predisposition phenotype | {Osteofibrous dysplasia, susceptibility to}, 607278 (3), i:AD; Hepatocellular carcinoma, childhood type, somatic, 114550 (3); ?Deafness, autosomal recessive 97, 616705 (3), i:AR; Renal cell carcinoma, papillary, 1, familial and somatic, 605074 (3) |
| *MTOR* | Proto-oncogene | Multisystem phenotype | Smith-Kingsmore syndrome, 616638 (3), i:AD; Focal cortical dysplasia, type II, somatic, 607341 (3) |
| *MYD88* | Proto-oncogene | Multisystem phenotype | Immunodeficiency 68, 612260 (3), i:AR; Macroglobulinemia, Waldenstrom, somatic, 153600 (3) |
| *NF1* | Tumour suppressor gene, fusion | Multisystem phenotype/Hereditary cancer predisposition phenotype | Neurofibromatosis-Noonan syndrome, 601321 (3), i:AD; Leukemia, juvenile myelomonocytic, 607785 (3), i:AD, i:SMu; Neurofibromatosis, familial spinal, 162210 (3), i:AD; Watson syndrome, 193520 (3), i:AD; Neurofibromatosis, type 1, 162200 (3), i:AD |
| *NFE2L2* | Oncogene, tumour suppressor gene | Multisystem phenotype | Immunodeficiency, developmental delay, and hypohomocysteinemia, 617744 (3), i:AD |
| *NOTCH1* | oncogene, TSG, fusion | Multisystem phenotype | Aortic valve disease 1, 109730 (3), i:AD; Adams-Oliver syndrome 5, 616028 (3), i:AD |
| *NRAS* | Proto-oncogene | Multisystem phenotype | Epidermal nevus, somatic, 162900 (3); Melanocytic nevus syndrome, congenital, somatic, 137550 (3); Schimmelpenning-Feuerstein-Mims syndrome, somatic mosaic, 163200 (3); Colorectal cancer, somatic, 114500 (3); ?RAS-associated autoimmune lymphoproliferative syndrome type IV, somatic, 614470 (3); Thyroid carcinoma, follicular, somatic, 188470 (3); Neurocutaneous melanosis, somatic, 249400 (3); Noonan syndrome 6, 613224 (3), i:AD |
| *PIK3CA* | Proto-oncogene | Multisystem phenotype | Ovarian cancer, somatic, 167000 (3); Colorectal cancer, somatic, 114500 (3); CLAPO syndrome, somatic, 613089 (3); Cowden syndrome 5, 615108 (3); Hepatocellular carcinoma, somatic, 114550 (3); Breast cancer, somatic, 114480 (3); Macrodactyly, somatic, 155500 (3); Keratosis, seborrheic, somatic, 182000 (3); Gastric cancer, somatic, 613659 (3); Megalencephaly-capillary malformation-polymicrogyria syndrome, somatic, 602501 (3); Nevus, epidermal, somatic, 162900 (3); CLOVE syndrome, somatic, 612918 (3); Nonsmall cell lung cancer, somatic, 211980 (3) |
| *PIK3CD* | N/A | Multisystem phenotype | Immunodeficiency 14, 615513 (3), i:AD |
| *PIK3R2* | Proto-oncogene | Multisystem phenotype | Megalencephaly-polymicrogyria-polydactyly-hydrocephalus syndrome 1, 603387 (3), i:AD |
| *PMS2* | Tumour suppressor gene | Hereditary cancer predisposition phenotype | Colorectal cancer, hereditary nonpolyposis, type 4, 614337 (3); Mismatch repair cancer syndrome 4, 619101 (3) |
| *PPP2R1A* | Tumour suppressor gene | Multisystem phenotype | Mental retardation, i:AD 36, 616362 (3), i:AD |
| *PTEN* | Tumour suppressor gene | Multisystem phenotype/Hereditary cancer predisposition phenotype | Prostate cancer, somatic, 176807 (3); {Glioma susceptibility 2}, 613028 (3); Cowden syndrome 1, 158350 (3), i:AD; Lhermitte-Duclos syndrome, 158350 (3), i:AD; Macrocephaly/autism syndrome, 605309 (3), i:AD; {Meningioma}, 607174 (3), i:AD |
| *PTPN11* | Proto-oncogene | Multisystem phenotype/Hereditary cancer predisposition phenotype | LEOPARD syndrome 1, 151100 (3), i:AD; Metachondromatosis, 156250 (3), i:AD; Noonan syndrome 1, 163950 (3), i:AD; Leukemia, juvenile myelomonocytic, somatic, 607785 (3) |
| *RAC1* | Proto-oncogene | Multisystem phenotype | Mental retardation, i:AD 48, 617751 (3), i:AD |
| *RAD50* | Tumour suppressor gene | Multisystem phenotype/Hereditary cancer predisposition phenotype | Nijmegen breakage syndrome-like disorder, 613078 (3) |
| *RAD51C* | Fusion | Hereditary cancer predisposition phenotype | {Breast-ovarian cancer, familial, susceptibility to, 3}, 613399 (3); Fanconi anemia, complementation group O, 613390 (3), i:AR |
| *RAF1* | Oncogene, fusion | Multisystem phenotype | LEOPARD syndrome 2, 611554 (3); Noonan syndrome 5, 611553 (3), i:AD; Cardiomyopathy, dilated, 1NN, 615916 (3), i:AD |
| *RB1* | Tumour suppressor gene | Hereditary cancer predisposition phenotype | Small cell cancer of the lung, somatic, 182280 (3); Bladder cancer, somatic, 109800 (3); Retinoblastoma, trilateral, 180200 (3), i:AD, i:SMu; Osteosarcoma, somatic, 259500 (3); Retinoblastoma, 180200 (3), i:AD, i:SMu |
| *RET* | Oncogene, fusion | Multisystem phenotype | Multiple endocrine neoplasia IIB, 162300 (3), i:AD; Pheochromocytoma, 171300 (3), i:AD; Multiple endocrine neoplasia IIA, 171400 (3), i:AD; Medullary thyroid carcinoma, 155240 (3), i:AD; {Hirschsprung disease, protection against}, 142623 (3), i:AD; Central hypoventilation syndrome, congenital, 209880 (3), i:AD; {Hirschsprung disease, susceptibility to, 1}, 142623 (3), i:AD |
| *RHOA* | Oncogene, tumour suppressor gene | Multisystem phenotype | Ectodermal dysplasia with facial dysmorphism and acral, ocular, and brain anomalies, somatic mosaic, 618727 (3) |
| *RIT1* | Proto-oncogene | Multisystem phenotype | Noonan syndrome 8, 615355 (3), i:AD |
| *RRAS2* | Proto-oncogene | Multisystem phenotype | Ovarian carcinoma (3); Noonan syndrome 12, 618624 (3), i:AD |
| *RUNX1* | oncogene, TSG, fusion | Hereditary cancer predisposition phenotype | Platelet disorder, familial, with associated myeloid malignancy, 601399 (3), i:AD; Leukemia, acute myeloid, 601626 (3), i:AD, i:SMu |
| *SDHA* | Tumour suppressor gene | Hereditary cancer predisposition phenotype | i:MT complex II deficiency, nuclear type 1, 252011 (3), i:AR; Cardiomyopathy, dilated, 1GG, 613642 (3), i:AR; Paragangliomas 5, 614165 (3), i:AD |
| *SDHAF2* | Tumour suppressor gene | Hereditary cancer predisposition phenotype | Paragangliomas 2, 601650 (3), i:AD |
| *SETD2* | Tumour suppressor gene | Multisystem phenotype | Luscan-Lumish syndrome, 616831 (3), i:AD |
| *SMAD3* | Tumour suppressor gene | Multisystem phenotype | Loeys-Dietz syndrome 3, 613795 (3), i:AD |
| *SMAD4* | Tumour suppressor gene | Multisystem phenotype/Hereditary cancer predisposition phenotype | Polyposis, juvenile intestinal, 174900 (3), i:AD; Juvenile polyposis/hereditary hemorrhagic telangiectasia syndrome, 175050 (3), i:AD; Myhre syndrome, 139210 (3), i:AD; Pancreatic cancer, somatic, 260350 (3) |
| *SMARCA4* | Tumour suppressor gene | Hereditary cancer predisposition phenotype | {Rhabdoid tumor predisposition syndrome 2}, 613325 (3), i:AD; Coffin-Siris syndrome 4, 614609 (3), i:AD |
| *SMARCB1* | Tumour suppressor gene | Multisystem phenotype/Hereditary cancer predisposition phenotype | Rhabdoid tumors, somatic, 609322 (3); {Schwannomatosis-1, susceptibility to}, 162091 (3), i:AD; Coffin-Siris syndrome 3, 614608 (3), i:AD; {Rhabdoid tumor predisposition syndrome 1}, 609322 (3), i:AD |
| *SMO* | Proto-oncogene | Multisystem phenotype | Curry-Jones syndrome, somatic mosaic, 601707 (3); Pallister-Hall-like syndrome, 241800 (3), i:AR; Basal cell carcinoma, somatic, 605462 (3) |
| *STAT3* | Proto-oncogene | Multisystem phenotype | Hyper-IgE recurrent infection syndrome, 147060 (3), i:AD; Autoimmune disease, multisystem, infantile-onset, 1, 615952 (3), i:AD |
| *STK11* | Tumour suppressor gene | Hereditary cancer predisposition phenotype | Testicular tumor, somatic, 273300 (3); Peutz-Jeghers syndrome, 175200 (3), i:AD; Melanoma, malignant, somatic, 155600 (3); Pancreatic cancer, somatic, 260350 (3) |
| *TGFBR1* | Oncogene(?) | Multisystem phenotype | Loeys-Dietz syndrome 1, 609192 (3), i:AD; {Multiple self-healing squamous epithelioma, susceptibility to}, 132800 (3), i:AD |
| *TGFBR2* | Tumour suppressor gene | Multisystem phenotype/Hereditary cancer predisposition phenotype | Esophageal cancer, somatic, 133239 (3); Colorectal cancer, hereditary nonpolyposis, type 6, 614331 (3); Loeys-Dietz syndrome 2, 610168 (3), i:AD |
| *TP53* | oncogene, TSG, fusion | Multisystem phenotype/Hereditary cancer predisposition phenotype | {Adrenocortical carcinoma, pediatric}, 202300 (3), i:AD; {Glioma susceptibility 1}, 137800 (3), i:AD, i:SMu; {Basal cell carcinoma 7}, 614740 (3), i:AD; Bone marrow failure syndrome 5, 618165 (3), i:AD; {Colorectal cancer}, 114500 (3), i:AD, i:SMu; Nasopharyngeal carcinoma, somatic, 607107 (3); Breast cancer, somatic, 114480 (3); {Osteosarcoma}, 259500 (3), i:SMu; {Choroid plexus papilloma}, 260500 (3), i:AD; Li-Fraumeni syndrome, 151623 (3), i:AD; Hepatocellular carcinoma, somatic, 114550 (3); Pancreatic cancer, somatic, 260350 (3) |
| *TP63* | Oncogene, tumour suppressor gene | Multisystem phenotype | Limb-mammary syndrome, 603543 (3), i:AD; Orofacial cleft 8, 618149 (3); Split-hand/foot malformation 4, 605289 (3), i:AD; Hay-Wells syndrome, 106260 (3), i:AD; Ectrodactyly, ectodermal dysplasia, and cleft lip/palate syndrome 3, 604292 (3), i:AD; Rapp-Hodgkin syndrome, 129400 (3), i:AD; ADULT syndrome, 103285 (3), i:AD |
| *VHL* | Tumour suppressor gene | Hereditary cancer predisposition phenotype | Pheochromocytoma, 171300 (3), i:AD; Erythrocytosis, familial, 2, 263400 (3), i:AR; von Hippel-Lindau syndrome, 193300 (3), i:AD; Renal cell carcinoma, somatic, 144700 (3); Hemangioblastoma, cerebellar, somatic (3) |

**Table B.** Genes from Cancer Hotspots with known modes of inheritance for associated Mendelian disease(s), including somatic and germline mechanisms of action and concordance status (concordant, discordant, or semi-concordant).

| **Gene** | **Cancer Mechanism** | **Mendelian Mechanism** | **Concordance** |
| --- | --- | --- | --- |
| *ACVR1* | GoF | GoF | Concordant |
| *AKT1* | GoF | GoF | Concordant |
| *AKT3* | GoF | GoF, LoF | Semi-concordant |
| *ALK* | GoF | GoF | Concordant |
| *ANKRD11* | LoF | LoF | Concordant |
| *APC* | LoF | LoF | Concordant |
| *AR* | GoF | GoF | Concordant |
| *ARAF* | GoF | GoF | Concordant |
| *ARID1A* | LoF | LoF | Concordant |
| *ARID1B* | LoF | LoF | Concordant |
| *ARID2* | LoF | LoF | Concordant |
| *ASXL2* | LoF | LoF | Concordant |
| *ATM* | LoF | LoF | Concordant |
| *AXL* | LoF | LoF | Concordant |
| *BCL10* | LoF | LoF | Concordant |
| *BCL2* | GoF | GoF, LoF | Semi-concordant |
| *BCL2L11* | GoF | GoF | Concordant |
| *BCOR* | LoF | LoF | Concordant |
| *BRAF* | GoF | GoF | Concordant |
| *BRCA2* | LoF | LoF | Concordant |
| *BRD4* | GoF | LoF | Discordant |
| *CARD11* | GoF | GoF, LoF | Semi-concordant |
| *CASP8* | LoF | LoF | Concordant |
| *CBL* | GoF, LoF | GoF | Semi-concordant |
| *CDH1* | LoF | LoF | Concordant |
| *CDK12* | LoF | LoF | Concordant |
| *CDK4* | GoF | GoF | Concordant |
| *CDKN1B* | LoF | LoF | Concordant |
| *CDKN2A* | LoF | LoF | Concordant |
| *CHEK2* | LoF | Lof | Concordant |
| *CIC* | GoF, LoF | LoF | Semi-concordant |
| *CREBBP* | GoF, LoF | GoF, LoF | Concordant |
| *CRLF2* | GoF | GoF | Concordant |
| *CTCF* | LoF | LoF | Concordant |
| *CTLA4* | GoF | LoF | Discordant |
| *CTNNB1* | GoF | LoF | Discordant |
| *CUL3* | LoF | LoF | Concordant |
| *CYSLTR2* | GoF | GoF | Concordant |
| *DICER1* | LoF | LoF | Concordant |
| *DNMT3A* | LoF | GoF, LoF | Semi-concordant |
| *DNMT3B* | LoF | LoF | Concordant |
| *EGFR* | GoF | LoF | Discordant |
| *EIF1AX* | GoF | GoF | Concordant |
| *EP300* | LoF | LoF | Concordant |
| *EPHA7* | LoF | LoF | Concordant |
| *ERBB2* | GoF | LoF | Discordant |
| *ERBB3* | GoF | LoF | Discordant |
| *ERBB4* | GoF, LoF | GoF, LoF | Concordant |
| *ERRFI1* | LoF | LoF | Concordant |
| *ESR1* | GoF, LoF | LoF | Semi-concordant |
| *ETV6* | LoF | LoF | Concordant |
| *EZH2* | GoF, LoF | GoF, LoF | Concordant |
| *FAT1* | LoF | LoF | Concordant |
| *FGFR1* | GoF | LoF | Discordant |
| *FGFR2* | GoF | GoF | Concordant |
| *FGFR3* | GoF | GoF, LoF | Semi-concordant |
| *FGFR4* | GoF | GoF | Concordant |
| *FH* | LoF | LoF | Concordant |
| *FOXL2* | GoF, LoF | LoF | Semi-concordant |
| *FOXP1* | GoF | LoF | Discordant |
| *FUBP1* | GoF | LoF | Discordant |
| *GATA2* | GoF | LoF | Discordant |
| *GATA3* | GoF, LoF | LoF | Semi-concordant |
| *GLI1* | GoF | LoF | Discordant |
| *GNA11* | GoF | GoF | Concordant |
| *GNAS* | GoF | LoF, GoF | Semi-concordant |
| *GTF2I* | GoF | GoF | Concordant |
| *H3-3A* | GoF | LoF | Discordant |
| *H3C2* | GoF | GoF | Concordant |
| *HNF1A* | LoF | LoF | Concordant |
| *HRAS* | GoF | GoF | Concordant |
| *IDH2* | GoF | GoF | Concordant |
| *IKZF1* | LoF | LoF | Concordant |
| *IL7R* | GoF | LoF | Discordant |
| *INPPL1* | LoF | LoF | Concordant |
| *JAK1* | GoF, LoF | GoF, LoF | Concordant |
| *KDM6A* | GoF, LoF | LoF | Semi-concordant |
| *KDR* | GoF | LoF | Discordant |
| *KEAP1* | LoF | LoF | Concordant |
| *KIT* | GoF | GoF, LoF | Semi-concordant |
| *KLF4* | GoF, LoF | GoF, LoF | Concordant |
| *KMT2C* | LoF | LoF | Concordant |
| *KMT2D* | GoF, LoF | GoF, LoF | Concordant |
| *KNSTRN* | GoF | LoF | Discordant |
| *KRAS* | GoF | GoF | Concordant |
| *MAP2K1* | GoF | GoF | Concordant |
| *MAP2K2* | GoF | GoF | Concordant |
| *MAP3K1* | GoF, LoF | GoF, LoF | Concordant |
| *MAPK1* | GoF | GoF | Concordant |
| *MED12* | LoF | LoF | Concordant |
| *MST1* | LoF | GoF | Discordant |
| *MTOR* | GoF | GoF | Concordant |
| *MYCN* | GoF | LoF | Discordant |
| *MYD88* | GoF | LoF | Discordant |
| *MYOD1* | GoF | LoF | Discordant |
| *NF1* | LoF | LoF | Concordant |
| *NFE2L2* | GoF, LoF | GoF, LoF | Concordant |
| *NOTCH1* | GoF, LoF | GoF, LoF | Concordant |
| *NOTCH2* | GoF, LoF | GoF, LoF | Concordant |
| *NRAS* | GoF | GoF | Concordant |
| *PAX5* | GoF, LoF | LoF | Semi-concordant |
| *PIK3CA* | GoF | GoF | Concordant |
| *PIK3R1* | LoF | GoF | Discordant |
| *PIK3R2* | GoF | GoF | Concordant |
| *PIM1* | GoF | GoF | Concordant |
| *PMS2* | LoF | LoF | Concordant |
| *POLE* | LoF | LoF | Concordant |
| *PPP2R1A* | LoF | GoF | Discordant |
| *PPP6C* | LoF | LoF | Concordant |
| *PTEN* | LoF | LoF | Concordant |
| *PTPN11* | GoF | GoF | Concordant |
| *PTPRT* | LoF | LoF | Concordant |
| *RAC1* | GoF | GoF, LoF | Semi-concordant |
| *RAD50* | LoF | LoF | Concordant |
| *RAF1* | GoF | GoF | Concordant |
| *RARA* | GoF | GoF | Concordant |
| *RB1* | LoF | LoF | Concordant |
| *RBM10* | LoF | LoF | Concordant |
| *RET* | GoF | GoF | Concordant |
| *RIT1* | GoF | GoF | Concordant |
| *RNF43* | LoF | LoF | Concordant |
| *RPS6KA4* | LoF | LoF | Concordant |
| *RUNX1* | GoF, LoF | GoF, LoF | Concordant |
| *SDHA* | LoF | LoF | Concordant |
| *SDHAF2* | LoF | LoF | Concordant |
| *SESN2* | GoF, LoF | GoF, LoF | Concordant |
| *SETD2* | LoF | LoF | Concordant |
| *SMAD2* | LoF | LoF | Concordant |
| *SMAD3* | LoF | GoF, LoF | Semi-concordant |
| *SMAD4* | LoF | GoF | Discordant |
| *SMARCA4* | LoF | GoF | Discordant |
| *SMARCB1* | LoF | GoF | Discordant |
| *SMARCD1* | LoF | LoF | Concordant |
| *SMO* | GoF | LoF | Discordant |
| *SOCS1* | LoF | LoF | Concordant |
| *SOS1* | GoF | GoF | Concordant |
| *SOX17* | LoF | LoF | Concordant |
| *SPOP* | LoF | GoF | Discordant |
| *SPRED1* | LoF | LoF | Concordant |
| *STAG2* | LoF | LoF | Concordant |
| *STAT3* | GoF | GoF | Concordant |
| *STK11* | LoF | LoF | Concordant |
| *SUZ12* | GoF, LoF | LoF | Semi-concordant |
| *TBX3* | GoF, LoF | LoF | Semi-concordant |
| *ELOC* | LoF | LoF | Concordant |
| *TCF3* | GoF, LoF | LoF | Semi-concordant |
| *TGFBR1* | GoF | GoF, LoF | Semi-concordant |
| *TGFBR2* | LoF | LoF | Concordant |
| *TNFRSF14* | LoF | LoF | Concordant |
| *TP53* | GoF, LoF | LoF | Semi-concordant |
| *TP63* | GoF, LoF | GoF, LoF | Concordant |
| *U2AF1* | GoF | GoF | Concordant |
| *VHL* | LoF | LoF | Concordant |
| *XPO1* | GoF | GoF | Concordant |

Table C. Odds ratio scores for classifying germline missense variants in ClinVar and their overlap with cancer mutations in Cancer Hotspots.

| Variant Classification in ClinVar | Present in Cancer Hotspots | Absent in Cancer Hotspots | OR*^a^* | CI*^b^* |
| --- | --- | --- | --- | --- |
| LP/P | 426 | 2723 | — | — |
| LB/B | 4 | 2751 | 107.6^***^ | 40.1-288.4 |
| VUS | 261 | 45181 | — | — |
| LB/B/VUS | 265 | 47932 | 28.3^***^ | 24.2-33.1 |
| LB/B/VUS/CIP | 379 | 50870 | 21.0^***^ | 18.2-24.2 |

*^a^* The three odds ratios (OR) compare likely pathogenic (LP)/pathogenic (P) variants with likely benign (LB)/benign (B) variants, LB/B variants/variants of uncertain significance (VUS), and LB/B variants/VUS/conflicting interpretations of pathogenicity (CIP) variants.

*^b^* 95% confidence interval (CI)

^***^ p<0.001

Table D. Positive likelihood ratios for classifying germline missense variants in ClinVar and their overlap with cancer mutations in Cancer Hotspots.

| Present in Cancer Hotspots | LP/P in ClinVar | LB/B in ClinVar | LR+^a^ |
| --- | --- | --- | --- |
| Yes | 426 | 4 | 93.2 |
| No | 2723 | 2751 |  |
|  | **LP/P in ClinVar** | **LB/B/VUS in ClinVar** |  |
| Yes | 426 | 265 | 24.6 |
| No | 2723 | 47932 |  |
|  | **LP/P in ClinVar** | **LB/B/VUS/CIP in ClinVar** |  |
| Yes | 426 | 379 | 18.3 |
| No | 2723 | 50870 |  |

^a^Positive likelihood ratio (LR+) comparing likely pathogenic (LP)/pathogenic (P) variants with likely benign (LB)/benign (B) variants, LB/B variants + variants of uncertain significance (VUS), and LB/B variants + VUS + conflicting interpretations of pathogenicity (CIP) variants. See Supplemental Methods for additional details.

**Table E.** Variants from controlled-access databases that overlapped with cancer mutations from Cancer Hotspots, along with participant counts and ClinVar classifications.

|  | Total participants sequenced*^a^* | Total participants with matches^b^ | Total variant  matches | Variant classification in ClinVar*^c^* | | | |
| --- | --- | --- | --- | --- | --- | --- | --- |
|  |  |  |  | LP/P | LB/B | VUS | N/P |
| GEL | 1,048,576 | 334 | 144 | 46 | 1 | 29 | 68 |
| MSSNG | 11,312 | 21 | 15 | 3 | 0 | 8 | 4 |
| G4RD | 2,799 | 24 | 17 | 3 | 0 | 2 | 12 |
| GeneDx | 400,000 | 1,296 | 175 | 111 | 0 | 27 | 37 |
| Total | 1,462,687 | 1,675 | 351 | 163 | 1 | 66 | 121 |
| **Unique** | **1,462,687** | **1,675** | **302** | **140** | **1** | **53** | **108** |

*^a^*Total number of participants in the queried database.

*^b^*Participants with variants overlapping cancer mutations from Cancer Hotspots.

*^c^*Overlapping variants with ClinVar information: LP/P, LB/B, VUS, N/P (absent in ClinVar).
