# Supplementary material for: Leveraging cancer mutation data to inform the pathogenicity classification of germline missense variants
Source: PLoS Genet. 2025 Jan 6;21(1):e1011540. doi: 10.1371/journal.pgen.1011540 (PMC11737861; doi:10.1371/journal.pgen.1011540)
Supplement: S3 Text — (DOCX) [file pgen.1011540.s003.docx]

**SUPPLEMENTAL FILE FOR:** Leveraging cancer mutation data to inform the pathogenicity classification of germline missense variants

**TABLE OF CONTENTS**

Supplemental Appendix 1 ..……………………………………………………………...….…2

Supplemental Appendix 2 ..……………………………………………………………...….…5

**Supplemental Appendix 1.** R script used to train and test logistic regression model.

# Load necessary libraries

library(ggplot2)

library(PRROC)

# Load and preprocess the training data

df <- read.csv("your_file_name.csv", header = TRUE)

# Filter data to exclude rows where REVEL > 0.290 for VUS classification

df <- df[!(df$clinvar_class == "VUS" & df$REVEL > 0.290), ]

# Generate a list of genes associated with cancer

genes_list <- unique(df$gene[df$cancer_binary == "1"])

# Subset df to include only genes in genes_list

df <- df[df$gene %in% genes_list, ]

# Convert specific columns to factors

factor_columns <- c("gene", "gene_residue", "residue", "cancer_binary", "outcome_binary", "clinvar_class", "allele_categorical")

df[factor_columns] <- lapply(df[factor_columns], as.factor)

# Remove rows with missing values in key predictive columns

df <- df[complete.cases(df[, c("phyloP7way_vertebrate", "phastCons7way_vertebrate", "REVEL")]), ]

# Build logistic regression model to predict binary outcome

model <- glm(outcome_binary ~ gene + residue_count + cancer_binary + cancer_count +

phyloP7way_vertebrate + phyloP20way_mammalian +

phastCons7way_vertebrate + phastCons20way_mammalian,

family = "binomial", data = df, weights = df$wgts)

# Summarize model results without scientific notation

options(scipen = 999)

summary(model)

# Load and preprocess the test dataset

dfTest <- read.csv("your_file_name.csv")

# Function to convert data types for specified columns

convertDataType <- function(df, columns, type) {

df[columns] <- lapply(df[columns], type)

return(df)

}

# Convert categorical columns to factors and numerical columns to numeric

dfTest <- convertDataType(dfTest,

c("gene", "gene_residue", "residue", "cancer_binary", "clinvar_class"),

as.factor)

dfTest <- convertDataType(dfTest,

c("phyloP7way_vertebrate", "phyloP20way_mammalian",

"phastCons7way_vertebrate", "phastCons20way_mammalian",

"REVEL", "SIFT", "PolyPhen2", "AlphaMissense"),

as.numeric)

# Replace "NA" strings with actual NA values and remove incomplete rows

dfTest[dfTest == "NA"] <- NA

dfTest <- dfTest[complete.cases(dfTest[c("phyloP7way_vertebrate", "phastCons7way_vertebrate")]), ]

# Predict outcome probabilities on the test dataset

test_pred_prob <- predict(model, newdata = dfTest, type = "response")

# Separate predicted probabilities by class (positive: outcome_binary=1, negative: outcome_binary=0)

scores.class0 <- test_pred_prob[dfTest$outcome_binary == "1"]

scores.class1 <- test_pred_prob[dfTest$outcome_binary == "0"]

# Plot Precision-Recall curve

prc <- pr.curve(scores.class0, scores.class1, curve = TRUE)

plot(prc, ylim = c(0, 1), xlim = c(0, 1), col = "#1F57C3", lwd = 3,

main = "Precision-Recall Curve", xlab = "Recall", ylab = "Precision")

**Supplemental Appendix 2.** R script used to train and test random forest model.

# Load necessary libraries

library(randomForest)

library(PRROC)

# Load and preprocess the training dataset

df <- read.csv("your_file_name.csv", header = TRUE)

# Filter data to exclude rows where REVEL > 0.290 for VUS classification

df <- df[!(df$clinvar_class == "VUS" & df$REVEL > 0.290), ]

# Generate a list of genes associated with cancer

genes_list <- unique(df$gene[df$cancer_binary == "1"])

# Subset df to include only genes in genes_list

df <- df[df$gene %in% genes_list, ]

# Convert specific columns to factors

df$cancer_binary <- as.factor(df$cancer_binary)

df$outcome_binary <- as.factor(df$outcome_binary)

# Remove rows with missing values in key predictive columns

df <- df[complete.cases(df[, c("phyloP7way_vertebrate", "phastCons7way_vertebrate")]), ]

# Build random forest model for binary outcome prediction

set.seed(42) # Ensures reproducibility

rf.model <- randomForest(outcome_binary ~ residue_count + cancer_binary + cancer_count +

phyloP7way_vertebrate + phyloP20way_mammalian +

phastCons7way_vertebrate + phastCons20way_mammalian,

data = df, mtry = 4, importance = TRUE, ntree = 350, proximity = FALSE)

# Load and preprocess the test dataset

dfTest <- read.csv("your_file_name.csv", header = TRUE)

# Convert relevant columns to factors in test data

factor_columns_test <- c("gene", "gene_residue", "residue", "cancer_binary", "clinvar_class", "outcome_binary")

dfTest[factor_columns_test] <- lapply(dfTest[factor_columns_test], as.factor)

# Remove rows with missing values in relevant columns and subset by genes_list

dfTest <- dfTest[complete.cases(dfTest[, c("phyloP7way_vertebrate", "phastCons7way_vertebrate", "REVEL")]), ]

dfTest <- dfTest[dfTest$gene %in% genes_list, ]

# Predict outcome probabilities on the test dataset using random forest model

test_pred_prob_rf <- predict(rf.model, dfTest, type = "prob")[, 2]

# Separate predicted probabilities by class (positive: outcome_binary=1, negative: outcome_binary=0)

scores.class0_rf <- test_pred_prob_rf[dfTest$outcome_binary == "1"]

scores.class1_rf <- test_pred_prob_rf[dfTest$outcome_binary == "0"]

# Plot Precision-Recall curve for random forest model

prc_rf <- pr.curve(scores.class0_rf, scores.class1_rf, curve = TRUE)

plot(prc_rf, ylim = c(0, 1), xlim = c(0, 1), col = "#8862CE", lwd = 3,

main = "Precision-Recall Curve", xlab = "Recall", ylab = "Precision")

lines(prc_rf$curve[,1], prc_rf$curve[,2], col = "#8862CE", lwd = 3)
