# Supplementary figures and images for: Leveraging cancer mutation data to inform the pathogenicity classification of germline missense variants
Source: PLoS Genet. 2025 Jan 6;21(1):e1011540. doi: 10.1371/journal.pgen.1011540 (PMC11737861; doi:10.1371/journal.pgen.1011540)

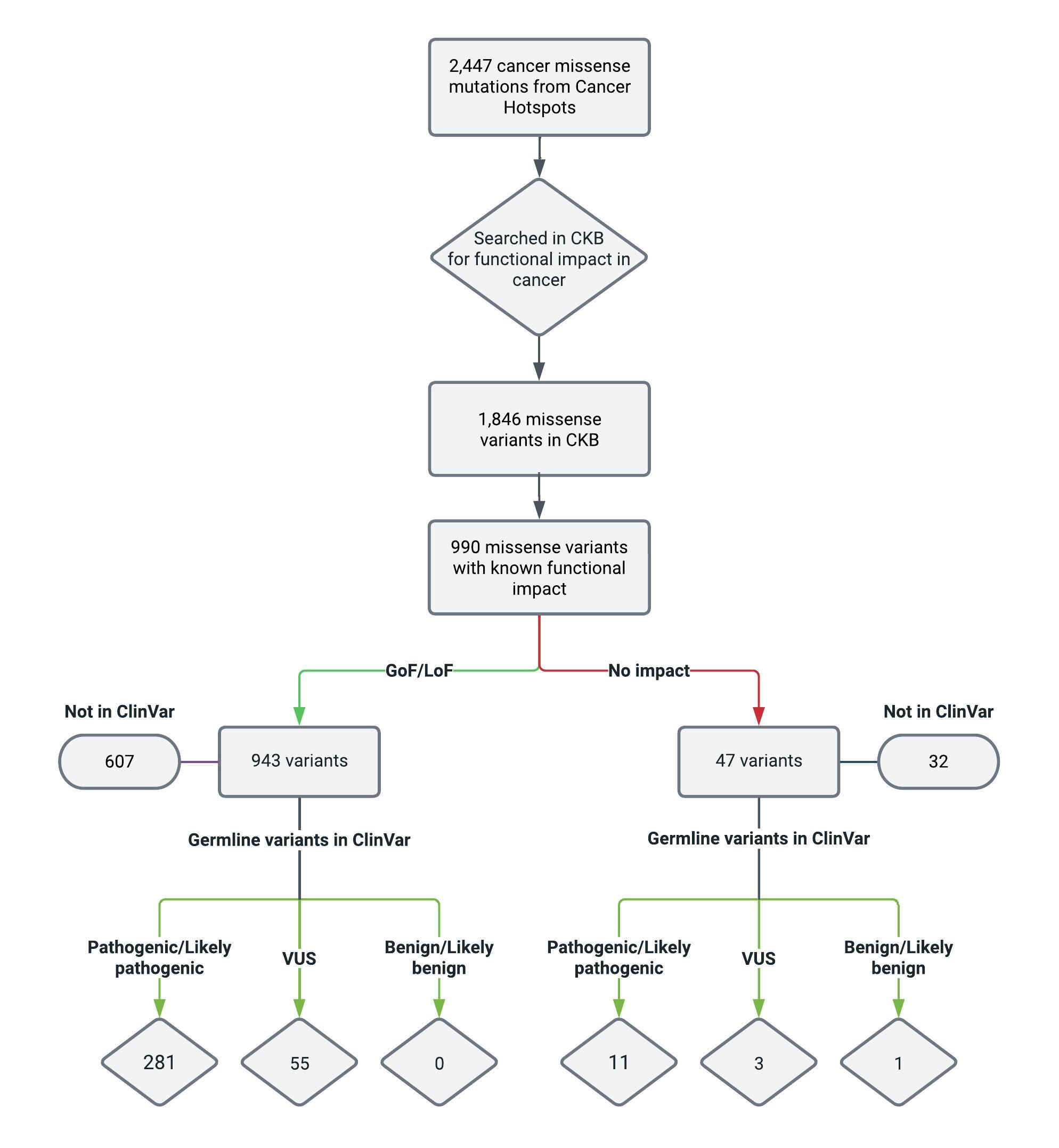

Supplement: S2 Fig — GoF, gain-of-function; LoF loss-of-function. (TIF) [file pgen.1011540.s006.tif]

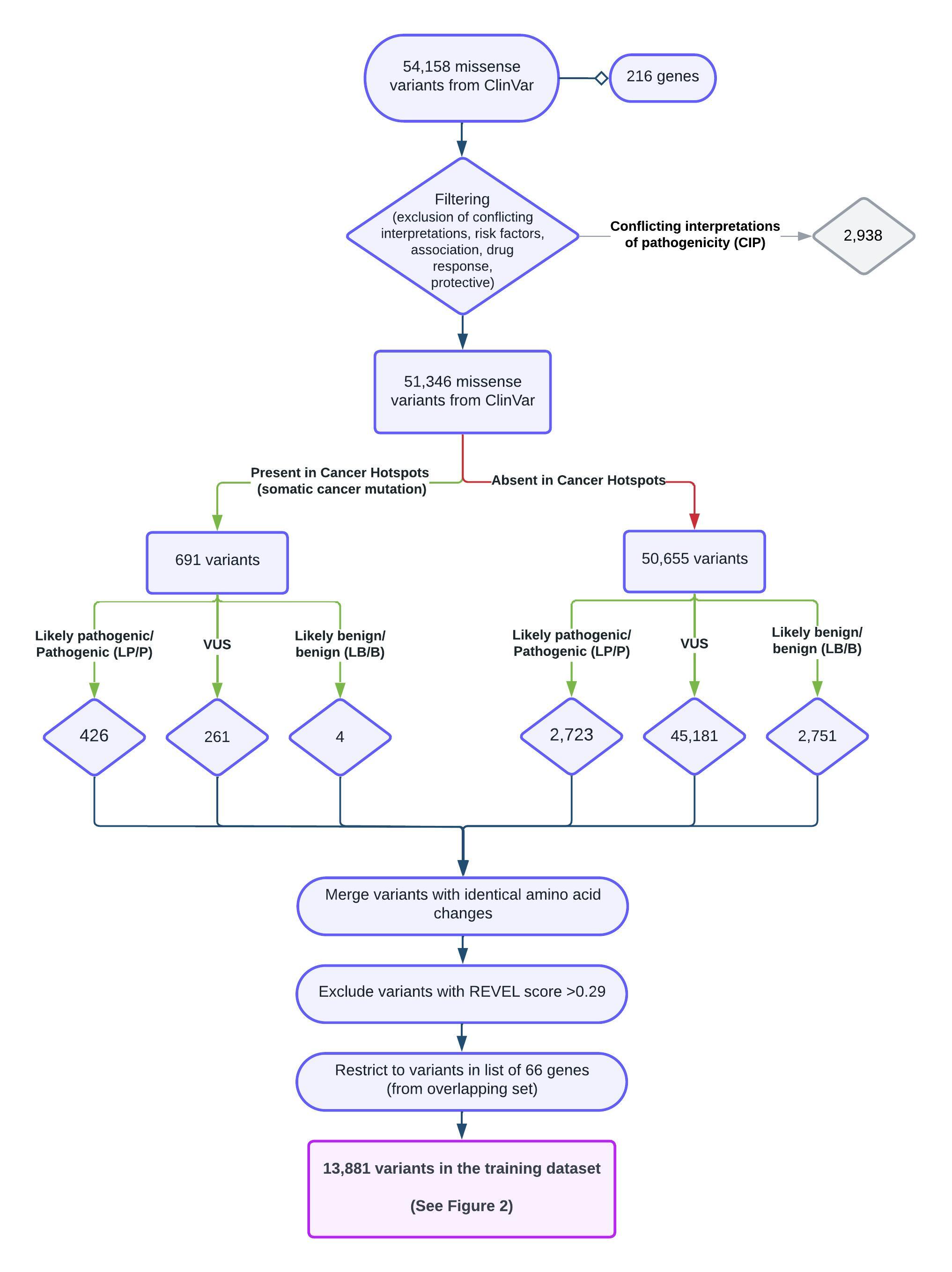

Supplement: S3 Fig — This illustrates the process of filtering the variants to create the “ClinVar dataset” used in the odd ratio calculations and as the training dataset for supervised learning models. (TIF) [file pgen.1011540.s007.tif]

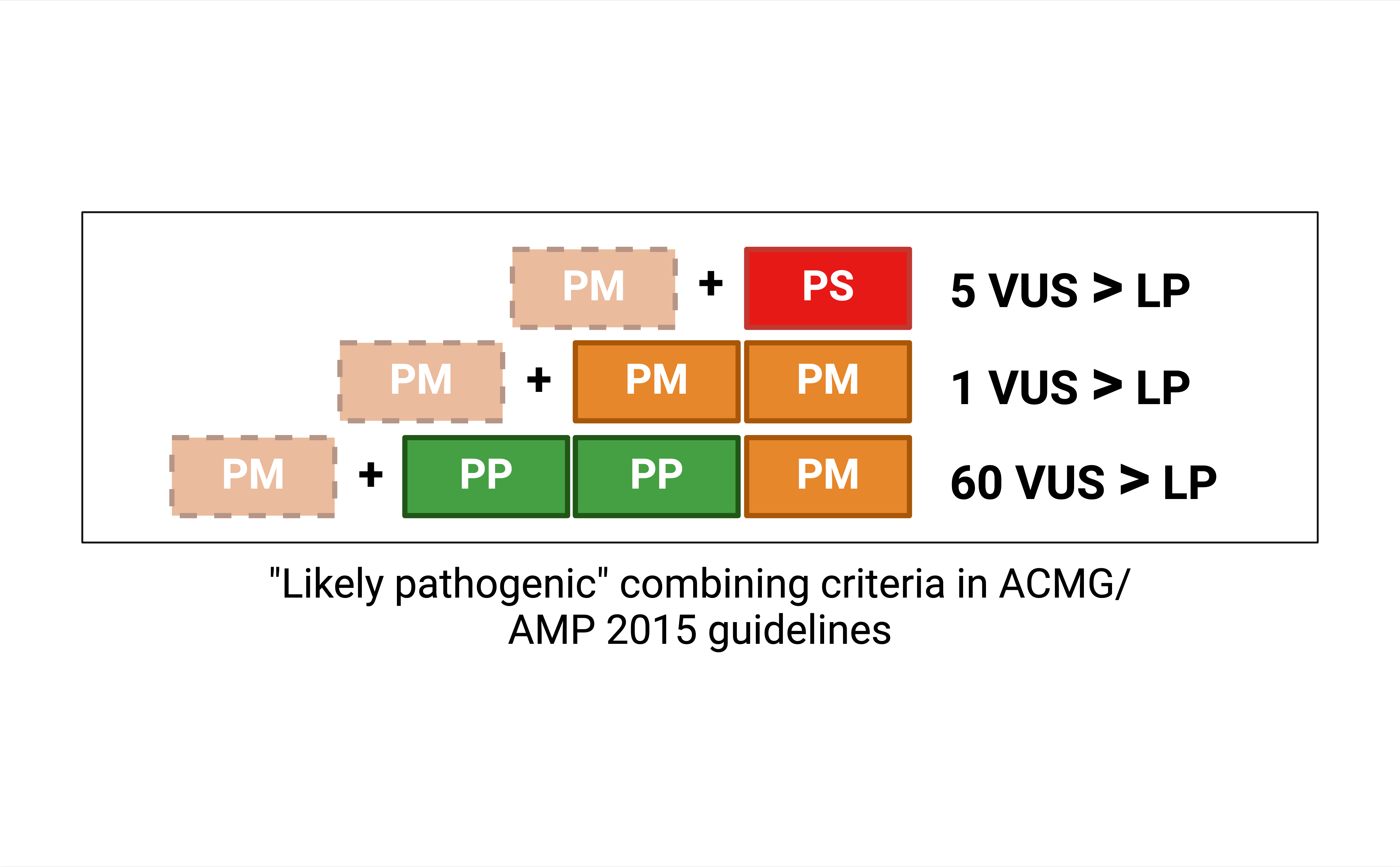

Supplement: S4 Fig — Each row represents the existing evidence codes for VUS, with the addition of one PM criterion, to form a combining criterion for the classification of “likely pathogenic” according to the ACMG/AMP guidelines. Among the 261 VUS, 12 were recently reclassified to LP/P (n = 11) or LB (n = 1) in ClinVar. With the remaining 249, an additional PM evidence code would be enough to potentially upgrade 66 VUS (26.5%) to LP. Figure was created with BioRender and adapted from Brnich et al., (2018) [2]. (TIF) [file pgen.1011540.s008.tif]

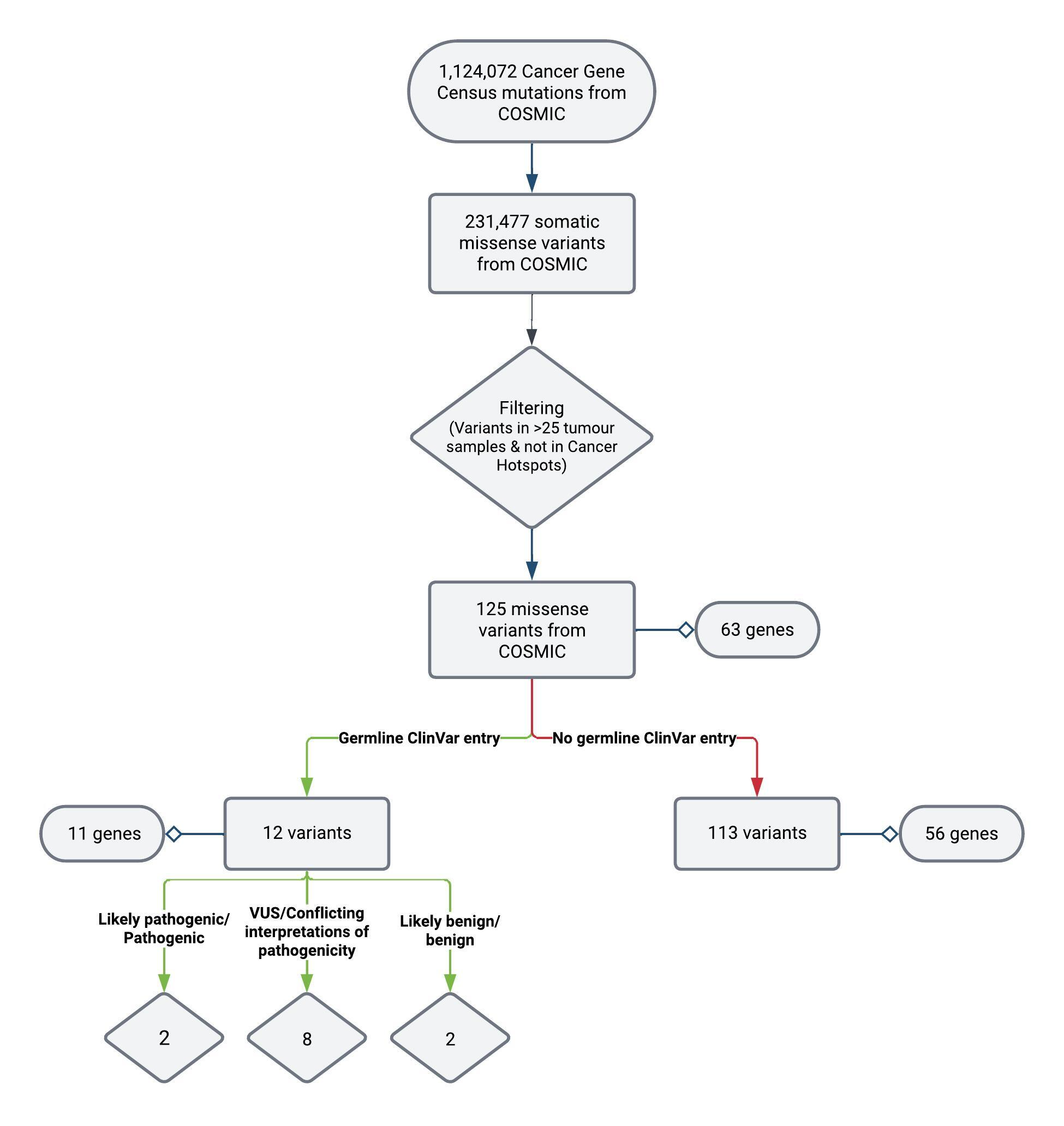

Supplement: S6 Fig — This figure illustrates the process of filtering COSMIC mutations using a stringent tumor sample count filter to identify additional cancer mutations that are absent from Cancer Hotspots. (TIF) [file pgen.1011540.s010.tif]

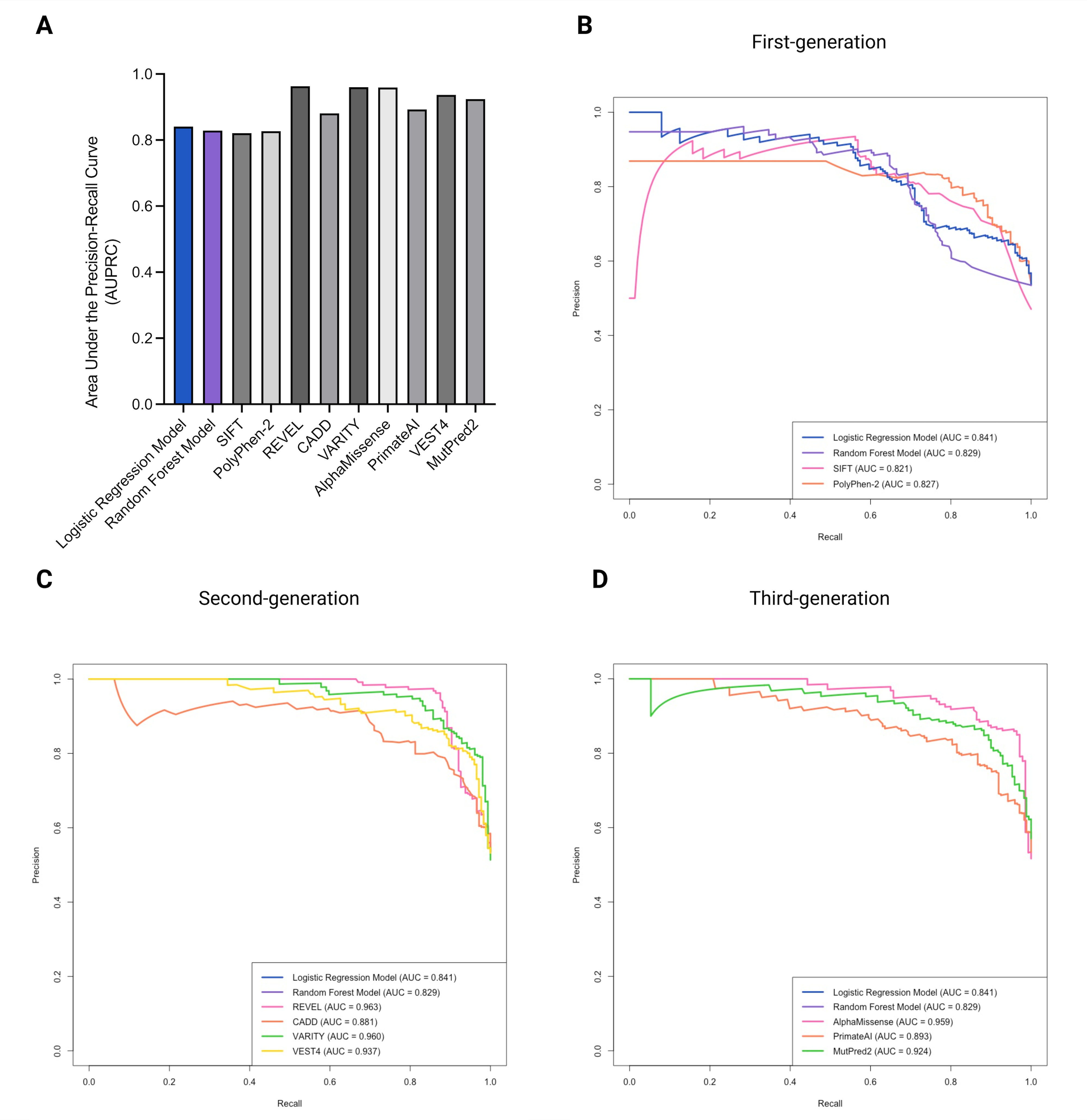

Supplement: S13 Fig — (A) Bar graph showing the area under the precision-recall curve (AUPRC) for each tool, including logistic regression model (LRM), random forest model (RFM), SIFT, PolyPhen-2, REVEL, CADD, VARITY, AlphaMissense, PrimateAI, VEST4, and MutPred2. (B) Precision-recall curves for first-generation tools (SIFT and PolyPhen-2) compared with LRM and RFM. (C) Precision-recall curves for second-generation tools (REVEL, CADD, VARITY, VEST4) compared with LRM and RFM. (D) Precision-recall curves for third-generation tools (AlphaMissense,PrimateAI, and MutPred2) compared with LRM and RFM. (TIF) [file pgen.1011540.s017.tif]
